# A case of late-onset, thymoma-associated myasthenia gravis with ryanodine receptor and titin antibodies and concomitant granulomatous myositis

**DOI:** 10.1186/s12883-016-0697-x

**Published:** 2016-09-13

**Authors:** M. I. Stefanou, L. Komorowski, S. Kade, A. Bornemann, U. Ziemann, M. Synofzik

**Affiliations:** 1 Department of Neurovascular Diseases, Hertie Institute for Clinical Brain Research & Center for Neurology, Tuebingen, Germany; 2 Institute for Experimental Immunology, Affiliated to Euroimmun AG, Luebeck, Germany; 3 Department of Neuropathology, University of Tuebingen, Tuebingen, Germany; 4 Department of Neurodegenerative Diseases, Hertie Institute for Clinical Brain Research & Center for Neurology, Tuebingen, Germany; 5 Deutsches Zentrum für Neurodegenerative Erkrankungen (DZNE), Tuebingen, Germany

**Keywords:** Myasthenia gravis, Ryanodine receptor antibodies, Titin antibodies, Granulomatous myositis

## Abstract

**Background:**

Myasthenia gravis is an autoimmune neuromuscular disorder, which has only rarely been reported to co-manifest with myositis. The diagnosis of concomitant myositis in patients with myasthenia gravis is clinically challenging, and requires targeted investigations for the differential diagnosis, including EMG, autoantibody assays, muscle biopsy and, importantly, imaging of the mediastinum for thymoma screening.

**Case presentation:**

This report presents a case-vignette of a 72-year-old woman with progressive proximal muscle weakness and myalgias, diagnosed with thymoma-associated myasthenia and bioptically verified granulomatous myositis, with positive autoantibody status for ryanodine receptor and titin antibodies.

**Conclusions:**

The diagnosis of concurrent myositis and myasthenia gravis, especially in the presence of ryanodine receptor and titin antibodies, should lead neurologists to adopt different treatment strategies compared to those applied in myasthenia or myositis alone. Moreover, further evidence is warranted that titin and, particularly, ryanodine receptor antibodies may co-occur or be pathophysiologically involved in myasthenia-myositis cases.

## Background

Myasthenia gravis (MG) is an organ-specific, autoimmune disorder, which is generally mediated by anti-acetylcholine receptor (AChR) or, less frequently, by anti-muscle-specific receptor tyrosine kinase (MuSK) antibodies (Ab) at the neuromuscular junction [1]. Striational-Ab, reacting with epitopes on muscle proteins and binding in a cross-striational pattern to skeletal and heart muscle tissue sections, have also been associated with severe, late-onset or thymoma-associated MG cases [2, 3]. Two major antigens for striational-Ab are: a) titin, a gigantic filamentous muscle protein, basically involved in muscle function, structure and development, and b) ryanodine receptor (RyR), a Ca^2+^ release channel of the sarcoplasmic reticulum, essential for the excitation-contraction coupling in striated muscle [4]. Titin-Ab and RyR-Ab are determined by enzyme-linked immunosorbent assay or immunoblot, and have been recently detected in first cases of thymoma-MG with co-manifesting myositis [5]. Although MG has been repeatedly associated with many autoimmune disorders, reports of patients with concomitant manifestation of MG-myositis are seldom [6–8]. Consequently, further evidence is warranted that titin-Ab and, particularly, RyR-Ab may be associated with MG-myositis cases.

## Case presentation

**Fig. 1:**
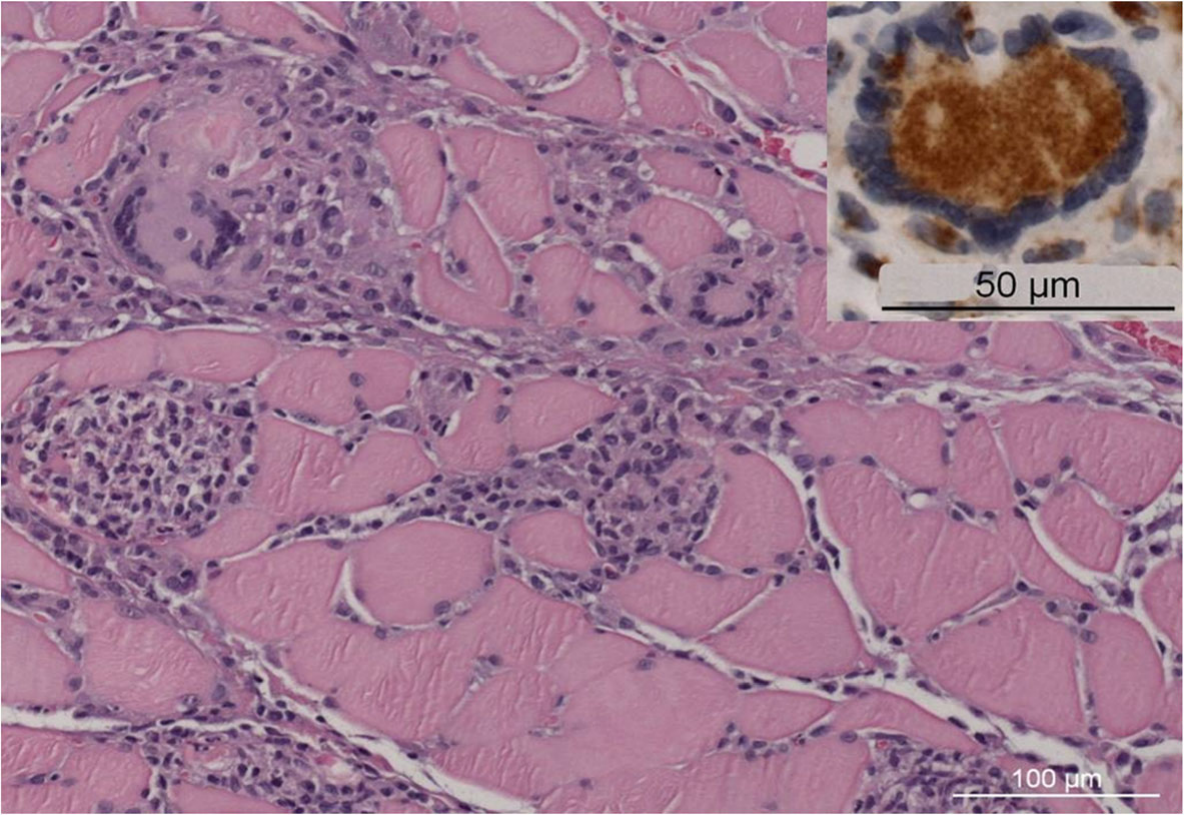
Granulomatous Myositis (deltoid muscle biopsy, hematoxylin and eosin staining). Abundant muscle infiltration with inflammatory cells and multinucleate giant cells. Inset: Giant cell expressing the macrophage marker CD68

A 72-year-old woman presented with 4 weeks of progressive, non-fluctuating, proximal muscle weakness and myalgia, after an acute manifestation of dysarthria. She complained of prominent fatigue and difficulty climbing stairs without assistance. In the family history no neurological disorders were reported. On physical examination, the patient had a moderate bilateral ptosis with positive test of eyelid fatigability, and a proximally accentuated muscle weakness in all extremities (grade 4/5 MRC in arm abductors, elbow extensors and hip flexors). Serological examination showed normal creatine kinase levels (70 U/l). Brain MRI presented no signs of cerebrovascular etiology of the acutely manifested dysarthria. Needle-EMG showed marked, pathological spontaneous activity (fibrillations and positive sharp waves) with complex, repetitive, pseudomyotonic discharges. In addition, muscle ultrasound revealed increased muscle echogenicity with prominent vascularization, suggestive of an active inflammatory myopathy. No decrement on 3Hz repetitive nerve stimulation was noted. A right deltoid muscle biopsy demonstrated endomysial CD68-immunoreactive giant cells and macrophages (Fig. 1), leading to the diagnosis of a granulomatous myositis (GrM). Furthermore, immunoassay analysis showed a high titer of AChR-Ab (142 nmol/l) and positive results for titin-Ab and RyR-Ab. No MuSK-Ab or other myositis-associated autoantibodies were detected. Malignancy screening, including chest CT, revealed a mediastinal mass suggestive of thymoma. In the chest CT scan, no pulmonary or mediastinal evidence of sarcoidosis was noted; additionally, the serum concentrations of angiotensin-converting enzyme (ACE) and soluble IL2 receptor (sIL2R) were normal. A video-assisted thoracoscopic thymectomy was performed, and pathological analysis disclosed a type AB, noninvasive (Masaoka stage I) thymoma. The patient was respectively diagnosed with thymoma-associated MG and GrM, and treatment with pyridostigmine, prednisone (initially 20 mg/d, increased to 60 mg/d after a week, and gradually tapered over 3 months), and azathioprine (75 mg/d) was initiated. Under the combined treatment regime, a marked improvement of her muscle strength was noted, with complete clinical remission after 3 months. Corresponding with this marked clinical improvement, repeated immunoassay analyses showed a progressive decline of AChR-Ab titer (23 nmol/l after 1 month, 7.55 nmol/l at 3 months), normalization of titin-Ab (negative at 3 months) and decline of RyR-Ab (lower reactivity in the immunoassay at 3 months).

## Conclusions

This report presents a case of histologically verified GrM and thymoma - associated MG with positive AChR-Ab, titin-Ab and RyR-Ab. GrM is a rare, inflammatory neuromuscular disorder, histologically characterized by the development of non-specific, epithelioid granulomas in striated muscle. Although a well-established association between GrM and sarcoidosis exists in the literature, GrM may accompany a wide spectrum of inflammatory and infectious diseases [9]. Interestingly, a concomitant manifestation of GrM with thymoma-MG is extremely rare [7, 8, 10]. The clinical hallmarks of GrM involve generalized muscle weakness, myalgias, and bulbar symptoms. Upon clinical suspicion of GrM, targeted procedures are warranted, including: a) investigation of muscular enzymes; these are commonly normal in the majority of GrM patients, yet, mild to high elevations have also been reported [9], b) imaging and laboratory investigations to assess muscle inflammatory processes, or to evaluate other organ affection when comorbid sarcoidosis is suspected (ACE and sIL2R were negative in this case), c) EMG testing, and d) pathological studies, which are mandatory for a definite diagnosis of GrM [9].

Conversely, in respect to the clinical manifestation, intermittent diplopia, ptosis and fluctuating muscle fatigability are classic features of MG, but are virtually never encountered in patients with myositis alone [6]. Given the partly overlapping symptoms of the two disorders, a co-manifesting GrM in patients with MG is likely to remain underdiagnosed. Yet, the presence of ‘red-flag’ features, such as a history of permanent, non-fluctuating muscle weakness, should lead physicians to consider comorbid myositis in MG patients. Imaging of the mediastinum is, importantly, recommended in all newly diagnosed cases of MG. Furthermore, electrophysiological studies, antibody assays, and muscle biopsy - even if the muscular enzymes are normal - are required for the differential diagnosis.

In particular, we suggest that myositis/MG -Ab investigations should include titin-Ab and RyR-Ab screening. Titin-Ab and RyR-Ab have a 70 % positive predictive value, and 95 % sensitivity and specificity for a thymoma in MG [4]. These striational antibodies have been repeatedly associated with thymoma-MG or severe, late-onset MG cases; nevertheless, their pathogenetic role in disease processes has, yet, not been clearly elucidated [11]. On the other hand, a broad spectrum of autoimmune disorders, and corresponding autoantibodies, have been related to thymoma [12]. The link between thymoma and autoimmunity probably indicates disrupted immunological surveillance mechanisms as a result of thymic dysfunction [12]. Thus, the occurrence of striational antibodies in thymoma-related GrM-MG cases may, to a certain extent, also reflect thymic dysfunction *per se* [12]. Additional evidence by future larger studies is warranted to investigate whether titin-Ab and RyR-Ab are frequently detected among MG-myositis patients, and whether the presence of these antibodies may suggest an underlying thymoma in the MG-myositis patient group.

Detecting the coincidence of MG and GrM is important, as in such cases different treatment strategies may be indicated. Considering that titin-Ab and RyR-Ab have been mostly associated with invasive/malignant types of thymoma, in the presence of such antibodies in MG or GrM-MG patients, the employment of a thymectomy technique that assures complete resection is decisive for the prognosis [4]. Lastly, given that in patients with MG - in contrast to myositis - initiation of therapy with high-dose steroids may exacerbate muscle weakness, in cases with concomitant manifestation of both disorders, a low-dose initiation of steroids with gradual titration, and concurrent administration of anticholinesterase agents, such as pyridostigmine, should be considered [6].

## Abbreviations

Ab: Antibodies
AChR: Anti-acetylcholine receptor
GrM: Granulomatous myositis
MG: Myasthenia gravis
MuSK: Anti-muscle-specific receptor tyrosine kinase
RyR: Ryanodine receptor
